# Psychosocial factors influencing quality of life in patients with primary brain tumors in Pakistan: an analytical cross-sectional study

**DOI:** 10.1186/s13104-023-06358-3

**Published:** 2023-05-25

**Authors:** Nida Zahid, Russell Seth Martins, Nick Brown, Wajeeha Zahid, Iqbal Azam, Aneesa Hassan, Khabir Ahmad, Shireen Shehzad Bhamani, Adnan Abdul Jabbar, Nargis Asad, Muhammad Shahzad Shamim, Rashid Jooma Khan, Gohar Javed, Ehsan Bari, Syed Ather Enam

**Affiliations:** 1 Department of Surgery, Aga Khan University Hospital, Karachi, Pakistan; 2 Center for Clinical Best Practice, Clinical and Translational Research Incubator, Aga Khan University Hospital, Stadium Road, Karachi, 74800 Pakistan; 3 Department of Women’s and Children’s Health, International Maternal and Child Health (IMCH), Uppsala University, Uppsala, Sweden; 4 Department of Community Health Sciences, Aga Khan University Hospital, Karachi, Pakistan; 5 School of Nursing and Midwifery, Aga Khan University Hospital, Karachi, Pakistan; 6 Department of Oncology, Aga Khan University Hospital, Karachi, Pakistan; 7 Department of Psychiatry, Aga Khan University Hospital, Karachi, Pakistan

**Keywords:** Quality of life, Anxiety, Depression, Social support, Developing Country, Brain tumor

## Abstract

**Objective:**

Despite quality of life (QoL) being recognized as an important outcome in neuro-oncology, there is a lack of research from Pakistan where sociocultural differences may influence QoL. This study aimed to measure the QoL in patients with primary brain tumors (PBTs) and assess its association with mental health outcomes and social support.

**Results:**

Our study included a total of 250 patients, with a median age of 42 years (range 33–54 years). The commonest brain tumors were glioma (46.8%) and meningioma (21.2). The mean global QoL of the sample was 75.73 ± 14.9. The majority of patients had high social support (97.6%) and were not depressed (90%) or anxious (91.6%).

On multivariable linear regression, global QoL was inversely associated with no or low income (beta coefficients: −8.75 to −11.84), having hypertension (−5.53), currently using a urine catheter (−13.55), having low social support (−28.16) suffering from mild (−15.31) or symptomatic (−23.84) depression, or mild anxiety (−13.22).

## Introduction

Patients with primary brain tumors (PBTs) often suffer from clinically significant psychological distress [1], the levels of which are higher than that experienced by patients suffering from most other types of malignancies [2]. Common mental health problems include depression, anxiety, stress, adjustment disorder and post-traumatic stress disorder, in addition to mental and emotional fatigue [2, 3, 4].

Quality of life (QoL) is an increasingly important outcome in clinical neuro-oncology that holistically encompasses functionality and wellness in the psychosocial, emotional, and physical domains [5]. Apart from the clinical and physical domains, existing literature suggests that QoL is majorly affected by psychosocial factors, including mental health, resilience, and social support. Mental health outcomes, particularly depression, correlate strongly with poorer QoL in patients with PBTs [6]. Social support, however, is associated with better QoL [7, 8].

The authors of the current paper have previously explored resilience, a quality that enables stable functionality through adversity, amongst patients with PBTs in Pakistan [9]. Greater resilience may help protect against adverse mental health outcomes in patients with PBTs, and thus improve QoL [10, 11].

There is a notable lack of research regarding the QoL of patients with PBTs in Pakistan [11]. Findings related to QoL, and its associated psychosocial factors, are likely to differ in Pakistan due to a unique economic, social, and cultural landscape [11]. A study in Pakistan conducted in 2020 showed that almost 40% of patients with PBTs have depressive symptoms, which were associated independently with social factors such as employment status [3]. As survivorship of patients with PBTs increases as a function of rapidly evolving therapies, QoL is being increasingly measured as an additional endpoint alongside traditional clinical outcomes in disease management and clinical trials [12]. There is an acutely increasing need to assess the QoL experienced by patients with PBT in Pakistan using widely accepted and standardized tools. The EORTC QLQ-C30 (European Organization for Research and Treatment of Cancer Quality of Life Questionnaire) and its brain tumor-specific module EORTC QLQ-BN20 (EORTC QLQ-Brain Neoplasms 20) have proved to be brief, reliable, and valid assessment measures in this regard [13, 14]. Thus, this study aimed to measure the QoL of patients with PBT in Pakistan and assess its association with mental health outcomes and social support.

## Main text

### Materials and methods

#### Study design and setting

This cross-sectional study was conducted at the Aga Khan University Hospital (AKUH), from November 2019 to May 2020. The protocol of this study has been approved by the institutional review board at AKUH and is published by the authors [11]. This paper is reported in accordance with the STROBE (strengthening the reporting of observational studies in epidemiology) checklist.

#### Study participants

We recruited all adult (≥ 18 years) patients treated for brain cancer at AKUH, provided that they gave informed consent and were currently ≥ 4 weeks post-initiation of treatment. We only considered patients who had been living in Pakistan for at least the past 3 months, as we wished to investigate the relationship of social support in the context of Pakistan with QoL. In addition, patients with any known pre-existing psychiatric history or on psychiatric medications, or with debilitating concurrent illnesses such as kidney failure or stroke, were excluded. However, patients were not excluded if they had hypertension, type 2 diabetes mellitus or cardiovascular disease, As the prevalence of these comorbids is relatively high in patients with brain cancer in Pakistan [15], excluding patients on the basis of these comorbids would have led to a nonrepresentative sample and hindered minimum sample size achievability.

#### Sampling technique

A minimum sample size of 250 was calculated. The assumptions of sample size are given elsewhere [9]. Nonprobability consecutive sampling was employed to recruit patients. Members of the research team screened all patients with brain cancer who were presenting to the neurosurgery or oncology clinics at AKUH for their scheduled appointment. If deemed eligible, patients were thoroughly briefed regarding the study and then requested for informed consent for participation. If patients agreed, they were administered the survey questionnaire by the data collector.

#### Data collection tools

The final questionnaire consisted of the following components:

- *Sociodemographic and Clinical Characteristics*: Participants’ sociodemographic details were recorded. Details are given elsewhere [9].
- *EORTC QLQ-C30*: This is a 30-item QOL measure for patients with cancer. It comprises of five functional scales (physical, role, cognitive, emotional, and social), three symptom scales (fatigue, pain, and nausea and vomiting), a global QOL scale, single items for measurement of other symptoms frequently experienced by cancer patients, and the perceived financial impact of the disease and treatment [13]. All items are scored using a 4-point Likert scale (1: ‘not at all’; to 4: ‘very much’), except for two items in the global QOL scale which instead employ 7- point linear analog scales. The functioning and global QOL subscales are scored ranging from 0 to 100, where higher scores imply favorable conditions. However, while symptom subscales are also scored ranging from 0 to 100, higher scores in these subscales imply greater symptoms i.e., unfavorable conditions.
- *EORTC QLQ-BN20*: This 20-item QOL measure is specifically for patients with primary brain neoplasms [14], and comprises four domains relevant to the disease (future uncertainty, visual disorder, motor dysfunction, and communication deficit), in addition to seven single items. All items are scored using a 4-point Likert scale (1: ‘not at all’; to 4: ‘very much’) and are then linearly converted to a 0–100 scale, where a higher score implies unfavorable conditions.
- *Psychosocial Characteristics*: The Hospital Anxiety and Depression Scale (HADS), a 14-item tool using a 4‐point ordinal scale, was used to measure patients’ depression and anxiety [11]. Patients’ social support was evaluated using the Enriched Social Support Instrument (ESSI) [11]. For depression and anxiety, the score obtained on HADS was categorized as follows: 0–7 (Normal); 8–10 (Mild Depression/Anxiety); and 11–21 (Symptomatic Depression/ Anxiety). A score ≤ 18 on the ESSI was classified as Low Social Support and > 18 as High Social Support.

Validated Urdu translations of EORTC QLQ-C30, EORTC BN20, HADS and ESSI, were used [16–19]. We have previously validated the HADS amongst patients with PBT in Pakistan and demonstrated excellent internal consistency for the overall tool (Cronbach’s alpha: 0.89) and for the depression (Cronbach’s alpha: 0.86) and anxiety (Cronbach’s alpha: 0.81) subscales [16]. The final Urdu questionnaire was pretested on 10% of the sample size to elucidate any ambiguities. However, no major changes were needed based on the pretest.

#### Statistical analysis

The data was analyzed by STATA version 15. Descriptive statistics for quantitative variables were reported as mean and standard deviation (SD) or median and range. For categorical variables, frequency and percentages were reported. The means and standard deviations of the QoL scales were calculated according to the EORTC QLQ-C30 manual. Linear regression was used to determine factors associated with global QoL, with unadjusted and adjusted beta coefficients, standard error (SE), and 95% confidence interval (CI) being reported. Interactions between all collected variables and global QoL were first assessed on univariate models. Variables with a p < 0.25 on univariate analysis were included in the multivariable linear regression model. A p < 0.05 was considered significant for all analyses.

## Results

### Study participants

Our study included 250 patients, with median age of 42 years (range 33–54 years) and 169/250 (67.6%) males. Urdu was the most common mother-tongue (30.8%), with Sindhi (18.8%) and Punjabi (14.8%) being others. Further details of patients’ sociodemographic characteristics are shown in Table 1 of the previous publication by the authors based on analysis of the same sample [9].

**Table 1:** Medical history and psychosocial characteristics

Variable	N (%)
Comorbids	
HTN	60 (24.0)
T2DM	37 (14.8)
CVD	4 (1.6)
Smoking status	
Current smoker	15 (6.0)
Ex-smoker	22 (8.8)
Never smoked	212 (85.2)
Smokeless tobacco use	
Current user	7 (2.8)
Ex-user	20 (8.0)
Never used	223 (89.2)
Tumor type	
Glioma	117 (46.8)
Meningioma	53 (21.2)
Schwannoma	12 (4.8)
Pituitary	44 (17.6)
Others	24 (9.6)
Surgical intervention	
Excisional biopsy	195 (78.0)
Total resection after diagnostic biopsy	11 (4.4)
Multiple interventions	27 (10.8)
No surgical interventions	17 (6.8)
Adjuvant therapy	
Chemotherapy	11 (4.4)
Radiotherapy	24 (9.6)
Combination	63 (25.2)
No adjuvant therapy	152 (60.8)
Status of cancer treatment	
Completed	112 (44.8)
Ongoing	138 (55.2)
Feeding tube needed	2 (0.8)
Tracheostomy needed	1 (0.4)
Urinary catheter needed	5 (2.0)
Social support	
≤ 18 (Low social support)	6 (2.4)
> 18 (High social support)	244 (97.6)
Depression	
0–7 (Normal)	225 (90.0)
8–10 (Mild depression)	14 (5.6)
11–21 (Symptomatic depression)	11 (4.4)
Anxiety	
0–7 (Normal)	229 (91.6)
8–10 (Mild anxiety)	16 (6.4)
11–21 (Symptomatic anxiety)	5 (2.0)

### Medical history and psychosocial characteristics

The commonest brain tumors were glioma (46.8%), meningioma (21.2%), and schwannoma (4.8%). Most patients underwent an excisional biopsy (78%), while 4.4% underwent total resection after prior diagnostic biopsy. Adjuvant therapy was received by 39.2% of patients. High social support was reported by most of the participants. Most of the participants were not depressed or anxious (Table 1).

### Quality of life

The mean global QoL score was 75.73. The values of the five functioning scales ranged from 81.2 (role functioning) to 87.93 (social functioning). In the symptom scales, pain had the highest score (29.7), followed by appetite loss (26.13), insomnia (25.06), and financial difficulties (21.06). The two worst symptoms on the BN-20 symptom scale were headache (21.46) and weakness (18.40). These are detailed in Table 2.

**Table 2:** Quality of Life (QoL) of Brain Tumor Patients as measured by the EORCT QLQ-C30 and QL-BN20

QLQ-C30 Component	Mean (SD)
Global QoL	75.73 (14.9)
Functional scale	
Physical functioning	81.65(21.45)
Role functioning	81.20 (33.02)
Emotional functioning	86.07 (13.34)
Cognitive functional	84.53 (20.39)
Social functional	87.93 (21.01)
Symptom scales	
Fatigue	9.55 (11.95)
Nausea	9.20 (15.00)
Pain	29.70 (30.48)
Dyspnea	4.66 (14.02)
Insomnia	25.06 (25.40)
Appetite loss	26.13 (26.40)
Constipation	2.13 (9.20)
Diarrhea	11.46 (22.59)
Financial difficulties	21.06 (32.59)

QLQ-BN20 component	Mean (SD)
Symptom scale	
Future uncertainty	7.53 (14.32)
Visual disorder	8.16 (15.30)
Motor symptoms	11.46 (17.15)
Communication deficit	9.33 (16.38)
Headache	21.46 (25.46)
Seizures	2.93 (11.96)
Drowsiness	10.13 (21.00)
Hair loss	10.53 (21.12)
Itchy skin	7.33 (17.27)
Weakness	18.40 (25.33)
Bladder control	5.86 (17.45)

### Linear regression analysis

On multivariable linear regression, having no income, an income of PKR 6000–25,000 or PKR 25,000–40,000, having hypertension, and currently using a urine catheter were significantly negatively associated with global QoL. Having low social support, suffering from mild or symptomatic depression, and mild anxiety were also associated with poorer QoL (Table 3).

**Table 3:** Univariate and multivariable linear regression with global QoL as dependent variable

Variables	Univariate analysis	Multivariable analysis
	Beta coefficient (SE) [95% CI]	Beta coefficient (SE) [95% CI]
Age (in years)	−0.12 (0.07) [−0.26, 0.01] *	NS
Formal schooling Yes (Reference) No	Reference −8.59 (3.36) [−15.22, −1.97] **	NS
Monthly family income (PKR/USD) No income 6000–25000/38–151 25000–40000/151–242 40000–80000/242–484 80000–1500000/484–1028 (Reference)	−9.06 (3.69) [−16.3, −1.78] ** −11.77 (2.70) [−17.10, −0.64] ** −6.17 (3.18) [−12.44, 0.08] * −3.56 (2.26) [−8.03,0.90] * Reference	−10.95 (3.09) [−17.04, −4.87] ** −11.84 (2.26) [−16.29, −7.38] ** −8.75 (2.66) [−13.99, −3.51] ** −3.88 (1.89) [−7.61, 0.16] Reference
Hypertension	−5.35 (2.18) [−9.66, −1.04] **	−5.53 (1.788) [−9.05, −2.02] **
T2DM	−4.56 (2.64) [−9.77, 0.65] *	NS
Smokeless tobacco use Current user Ex-user Never used (Reference)	−15.12 (6.480) [−27.88, −2.35] ** 0.36 (3.44) [−6.42, 7.15] Reference	NS
Status of cancer treatment Complete (reference) ongoing	Reference −2.44 (1.89) [−6.17, 1.28] *	NS
Using urine catheter	−22.85 (6.59) [−35.85, −9.87] **	−13.55 (5.64) [−24.66, −2.43] **
Social support ≤ 18 (Low social support) > 18 (High social support) (reference)	−14.98 (6.10) [−27.00, −2.95] ** Reference	−28.16 (13.35) [−1.84, −54.49] ** Reference
Depression 0–7 (normal) (reference) 8–10 (mild depression) 11–21 (symptomatic depression)	Reference −18.40 (3.68) [−25.66, −11.14] ** −26.41 (4.13) [−34.54, −18.27] **	Reference −15.31 (3.51) [−22.23, −8.39)] ** −23.84 (4.16) [−32.05, −15.64] **
Anxiety 0–7 (normal) (reference) 8–10 (Mild anxiety) 11–21 (symptomatic anxiety)	Reference −20.03 (3.62) [−27.16, −12.90] ** −15.6 (6.32) −28.12, −3.19] **	Reference −13.22 (3.24) [−19.61, −6.83] ** 0.34 (6.17) [−11.81, 12.51]

*SE* standard error, *CI* confidence interval

^*^ p < 0.2 on univariable analysis

^**^ p < 0.05

## Discussion

In our study, the mean global QoL (75.73) and scores on the five functional subscales (81.20–87.93) as measured by the EORTC QLQ-C30 are higher than a global weighted mean calculated using data from Austria, Germany, France, Turkey, Canada, The Netherlands, Iran and India [20]. In addition, all symptom components on the QLQ-BN20 were lower in our study, barring weakness of legs (mean of 18.40 ± 25.33 in our study vs. weighted mean 17.72 ± 92.99) [20]. It is possible that these findings of an overall better QoL in our sample are a function of the higher social support, and lower depression and anxiety, which contributes towards better QoL [9]. The higher social support, in turn, is likely due to the close-knit extended family systems that are a feature of South Asian households (54% of patients in our study lived in extended families). However, our study reported higher scores than the global weighted mean on 5/9 of the symptom scales on the QLQ-C30 (pain, insomnia, appetite loss, diarrhea, and financial difficulties). An interesting theory is that since the population of a developing country like Pakistan is generally accustomed to a lower standard of living and QoL than that of a developed country, the drop in QoL due to an illness may be perceived as less significant despite similar or worse symptomatology. Interestingly, the male predominance and median age of 42 years (range 33–54 years) represents the demographic that encompasses the breadwinners of most Pakistani families, which could explain the greater financial difficulties in our sample.

Comorbid hypertension, use of a urine catheter, lower family income, low social support, and coexisting anxiety and depression were associated with a poorer global QoL. These findings are self-explanatory and supported by previous literature. Hypertension, chronic need for a urinary catheter, and low socioeconomic status are known to be independently associated with poorer quality of life [21–23]. Greater social support [24] has been found to be associated with better QoL in patients with PBTs [8, 25], while poorer mental health, particularly anxiety and depression, is associated with worse QoL [26].

QoL assessment must become a routine feature of oncologic management for patients with PBTs in Pakistan, particularly at the initiation of treatment and then at regular intervals. Periodic screening for adverse mental health outcomes would allow for earlier identification and treatment, and the negation of subsequent harmful effects on QoL. Involvement of a psychologist in the routine care of all patients with PBTs could help prevent the development of mental ailments. Apart from this, since medical comorbids such as hypertension are also associated with poorer QoL, patients should be encouraged to maintain close follow-up with a primary care physician to optimize non-oncologic medical issues. In addition, since the current practice of using indwelling urinary catheters is also associated with a poorer QoL, clinicians may opt for clean intermittent self-catheterization as an alternate, as this is associated with better QoL [27]. Lastly, as lower income is an unfortunate and unavoidable risk factor for poorer QoL amongst patients with PBTs, and the primary mode of health payment in Pakistan is out-of-pocket, health systems should explore subsidized oncology packages and need-based financial aid for patients from lower socioeconomic backgrounds.

Future studies must aim to explore other patient and disease characteristics that may influence QoL in patients with PBTs, such as patient personality traits and biological tumor markers. The impact of personality traits on QoL in patients has been demonstrated in prior research amongst patients with breast [28], lung [29] and colorectal cancer [30]. This area of study is particularly relevant amongst patients with PBTs, as personality changes are a common feature of the disease. In addition, numerous cancer biomarkers such as cytokines, deoxyribonucleic acid (DNA), and ribonucleic acid (RNA) have been shown to be associated with patient-reported symptoms including pain, fatigue, depression, and sleep disturbances. Thus, it is plausible that biological tumor markers influence QoL, and these associations must be studied in the context of PBTs.

### Conclusions

The quality of life of patients with primary brain tumors in Pakistan is a function of clinical factors such as comorbid disease and use of a urinary catheter, social factors such a family income and social support, and psychological factors such as mental illness. Our findings may be of use in the development of QoL-improving interventions within the sociocultural setting of Pakistan.

### Limitations

The limitation of this study was its cross-sectional nature, which prevented us from establishing a temporal relationship between QoL and its associated psychological factors such as depression, and anxiety. In future, longitudinal studies should be conducted to evaluate QoL and its associated factors before treatment to the survival period, to better understand the relationship between QoL and mental health.

## Data Availability

The data that support the findings of this study are available on request from the corresponding author. The data is not publicly available due to privacy or ethical restrictions.

## Abbreviations

AKUH: Aga Khan University Hospital.
PBT: primary brain tumors
QoL: quality of life
EORTC QLQ-C30: European Organization for Research and Treatment of Cancer Quality of Life Questionnaire
EORTC QLQ-BN20: EORTC QLQ-Brain Neoplasms 20
STROBE: Strengthening the Reporting of Observational Studies in Epidemiology
HADS: Hospital Anxiety and Depression Scale
ESSI: Enriched Social Support Instrument
GLM-MANOVA: General linear model - multivariate analysis of variance
SE: standard error
CI: confidence interval
HTN: hypertension
T2DM: type 2 diabetes mellitus
PKR: Pakistani Rupee
DNA: Deoxyribonucleic acid
RNA: Ribonucleic acid
